# Comparison between an African town and a neighbouring village shows delayed, but not decreased, sleep during the early stages of urbanisation

**DOI:** 10.1038/s41598-017-05712-3

**Published:** 2017-07-18

**Authors:** Andrew D. Beale, Mario Pedrazzoli, Bruno da Silva B. Gonçalves, Felipe Beijamini, Núbia E. Duarte, Kieren J. Egan, Kristen L. Knutson, Malcolm von Schantz, Laura C. Roden

**Affiliations:** 1 Faculty of Health and Medical Sciences, University of Surrey, Guildford, Surrey UK; 2 School of Arts, Science, and Humanities, University of São Paulo, São Paulo, Brazil; 3 Federal University of Fronteira Sul, Realeza, Paraná Brazil; 4 Department of Mathematics, National University of Colombia, Manizales, Colombia; 5 Center for Circadian and Sleep Medicine, Department of Neurology, Northwestern University, Chicago, IL USA; 6 Department of Molecular & Cell Biology, University of Cape Town, Private Bag Rondebosch, Cape Town, South Africa

## Abstract

The well-established negative health outcomes of sleep deprivation, and the suggestion that availability of electricity may enable later bed times without compensating sleep extension in the morning, have stimulated interest in studying communities whose sleep pattern may resemble a pre-industrial state. Here, we describe sleep and activity in two neighbouring communities, one urban (Milange) and one rural (Tengua), in a region of Mozambique where urbanisation is an ongoing process. The two communities differ in the amount and timing of daily activity and of light exposure, with later bedtimes (≈1 h) associated with more evening and less daytime light exposure seen in the town of Milange. In contrast to previous reports comparing communities with and without electricity, sleep duration did not differ between Milange (7.28 h) and Tengua (7.23 h). Notably, calculated sleep quality was significantly poorer in rural Tengua than in Milange, and poor sleep quality was associated with a number of attributes more characteristic of rural areas, including more intense physical labour and less comfortable sleeping arrangements. Thus, whilst our data support the hypothesis that access to electricity delays sleep timing, the higher sleep quality in the urban population also suggests that some aspects of industrialisation are beneficial to sleep.

## Introduction

Urbanisation and industrialisation have led to multiple changes in lifestyle which are associated with an increased incidence of non-communicable diseases. Reduced sleep duration, attributed to artificial light at night and the use of electronic devices, has been suggested to be a contributing factor to this increase^1–3^. In an attempt to define the amount of sleep we are biologically adapted for, and which our ancestors may have enjoyed, some studies have investigated sleep in pre-industrialised communities that may display aspects of ‘ancestral’ or ‘natural’ sleep^4, 5^. Africans living in Africa have been of particular interest in this regard. Studies from North America have suggested ethnic differences in sleep and circadian biology between European- and African-Americans^6, 7^, but these differences are difficult to disentangle from the effects of admixture and socioeconomic factors. The ongoing urbanisation of many areas in sub-Saharan Africa affords us opportunities to study sleep in culturally and genetically similar populations in areas with varying degrees of urbanisation, including with and without access to electricity. Recent evaluations of sleep in hunter-gatherer societies (African and South American)^4, 5, 8^ and in Amazonian rubber tappers^9^ show that valuable insights can be gained from studying such communities. A recent study^4^ performed in undeveloped parts of Africa and South America showed sleep durations in three communities that were at the lower range of the averages reported in post-industrial societies. The interpretation of this observation has been controversial^10^, partly because of concerns that the daily rhythms observed in these hunter-gatherer communities are based on the need for survival rather than preference and/or wellbeing. Exploring sleep in low- and middle income countries beyond the increasingly rare hunter-gatherer societies, in a range of cultural and socioeconomic backgrounds, may be informative in determining changes in sleep associated with industrialisation. We have chosen to study a representative rural area of Southern Africa with some foci of ongoing urbanisation, with the aim of characterising and comparing sleep habits between two communities.

The district of Milange in the province of Zambézia, Mozambique, is one such location where culturally and ancestrally similar populations can be found living at different stages of urbanisation. According to the most recent census data, 7.4% of the population of the district was classed as urban and dwelling in the capital of the district, Milange, a small electrified market town^11^ (Supplementary Fig. S1). In 2007, 2% of the population of the district had access to electricity at home^12^, and between 2002 and 2008, access to electric light in the urban population grew from 18.7% to 34.8% of the urban population, demonstrating the rapid development of the town^13^. We recruited 74 permanent residents of the district of Milange, 37 of whom lived in the electrified town, Milange (16.10°S, 35.76°E), and 37 of whom lived in the neighbouring non-electrified rural community of Tengua (16.23°S, 35.81°E; see Supplementary Fig. S1 and Supplementary Fig. S2 for satellite images and geographical location of the region), in order to study the role of urbanisation in the timing of daily activity, the pattern of light exposure, and calculated measures of sleep.

## Results

### Daily activity and light exposure

**Figure 1:**
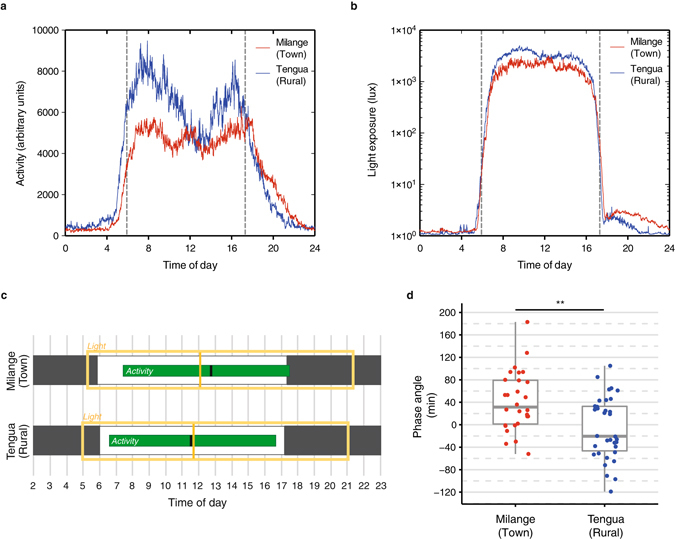
Daily activity and light exposure in rural (Tengua) and town (Milange) settings. Mean 24-h activity (**a**) and light exposure (**b**) profiles for all subjects of Tengua (blue) and Milange (red). Activity (arbitrary units) and light (lux) were measured in 60-s epochs by wrist-worn actigraphy devices. Dashed lines represent mean sunrise (05:55) and sunset (17:18) respectively. (**c**) Representation of the timing of daily activity and daily light exposure in Milange and Tengua based on non-parametric analysis of actigraphy. M10 activity (green bar), centre of gravity of activity (black line), M16 light exposure (yellow box), centre of gravity light exposure (yellow line). Black and white bars represent dark and light periods respectively in the natural environment. (**d**) The phase angle between the centres of gravity of activity and light exposure was significantly different in the two locations, with activity in Milange delayed relative to the light exposure. **P < 0.01.

The amount and timing of daytime activity and exposure to light during the day differed significantly between the two communities. Residents of rural Tengua showed significantly more activity in the most active ten-hour period (M10) than those of Milange (*n* = 62, Mann-Whitney-Wilcoxon, *W*(60) = 176, *P* < 0.001; Table 1), which corresponded to the demanding nature of their subsistence farming lifestyle. This difference was most striking in the early morning and late afternoon, which corresponded to morning and afternoon sessions of farming respectively (Fig. 1a). Between midday and 2 pm, the level of activity in Tengua reduced to a level similar to that found in Milange, described by residents as the period in which they return home from the farms to avoid the heat of midday. After sunset, Milange residents were more active than those of Tengua (*n* = 62, Student’s *t*-test, *t*(60) = 3.75, *P* < 0.001; Table 1 and Fig. 1a). During the day, Tengua residents were exposed to significantly more light than those of Milange (*n* = 62, Mann-Whitney-Wilcoxon, *W*(60) = 176, *P* < 0.001; Table 1 and Fig. 1b), with a reduction in exposure between midday and 2 pm (Fig. 1b) concurrent with the decrease in activity. An indication of entrainment to the external signal is given by the phase angle, which is the relationship between the timing of the circadian clock and the timing of an external time cue. In Tengua, the centres of gravity of activity (CG activity) and light exposure (CG light) were closely aligned (Table 1, Fig. 1c), and corresponded to the average solar noon during the course of the study (11:36). By contrast, the centre of gravity of activity of participants of Milange was delayed by 42 ± 10 min relative to the centre of gravity of light exposure, with the phase angle between activity and light significantly different between town (Milange) and rural (Tengua) communities (*n* = 62, Student’s *t*-test, *t*(60) = 3.44, *P* = 0.001; Fig. 1d).

**Table 1:** Activity, light, and sleep variables calculated from actigraphy.

Variables	Milange (Town)		Tengua (Rural)		p
	mean (n = 28)	SEM	mean (n = 34)	SEM	
M10 activity (AU)	3.3 × 10^6^	2.0 × 10^5^	4.4 × 10^6^	2.1 × 10^5^	5.92 × 10^−4 a^
L5 activity (AU)	4.4 × 10^4^	3.7 × 10^3^	7.1 × 10^4^	5.8 × 10^3^	2.47 × 10^−4 a^
M3 evening activity (AU)	5.3 × 10^5^	2.7 × 10^4^	3.9 × 10^5^	2.7 × 10^4^	4.02 × 10^−4^
M10 onset (24-h time)	7:29	0:18	6:36	0:10	0.013^b^
L5 onset (24-h time)	23:10	0:12	22:03	0:13	3.51 × 10^−4 a^
CG activity (24-h time)	12:47	0:11	11:35	0:10	1.18 × 10^−6 a^
M16 light (log lux)	6.04	0.03	6.24	0.03	9.60 × 10^−6 c^
M3 evening light (log lux)	2.54	0.03	2.39	0.02	1.63 × 10^−5 c^
CG light (24-h time)	12:06	0:05	11:41	0:04	8.11 × 10^−5 a^
IV	0.68	0.02	0.61	0.02	0.042^a^
IS	0.45	0.03	0.49	0.02	0.277^a^
Wake up time (24-h time)	5:54	0:08	5:21	0:05	3.63 × 10^−4 a^
Sleep onset (24-h time)	21:06	0:11	20:07	0:07	3.42 × 10^−6 c^
Total sleep period (h)	8.78	0.18	9.23	0.13	0.036^a^
Sleep duration (h)	7.28	0.18	7.23	0.17	0.860^a^
Sleep efficiency (%)	83.3	1.52	78.5	1.47	0.013^c^
WASO (min)	82.9	8.49	114.8	7.99	0.001^c^
Number of awakenings	13.2	0.90	16.3	0.96	0.024^a^

M10 = total activity in most active 10-hour period, L5 = total activity in least active 5-hour period, M3 evening activity = total activity in 3 hours post sunset, M10 onset = onset time of M10 period, L5 onset = onset time of L5 period, CG activity = centre of gravity of activity, M16 light = total light exposure in brightest 16 hour period, M3 evening light = total light exposure in 3 hours post sunset, CG light = centre of gravity of light exposure, IV = intradaily variability, IS = interdaily stability, total sleep period = total time of actigraphy-scored sleep period, sleep duration = total time asleep in actigraphy-scored sleep period, sleep efficiency = total sleep duration/total sleep period, WASO = wake after sleep onset, number of awakenings = number of actigraphy-scored awakenings in the sleep period. M10 onset, L5 onset, CG activity, CG light, wake up time and sleep onset are indicated according to clock time. AU = arbitrary units. ^a^Student’s *t*-test, ^b^Welch Two Sample *t*-test, ^c^Mann-Whitney *U* test.

### Amount and effect of artificial light exposure

**Figure 2:**
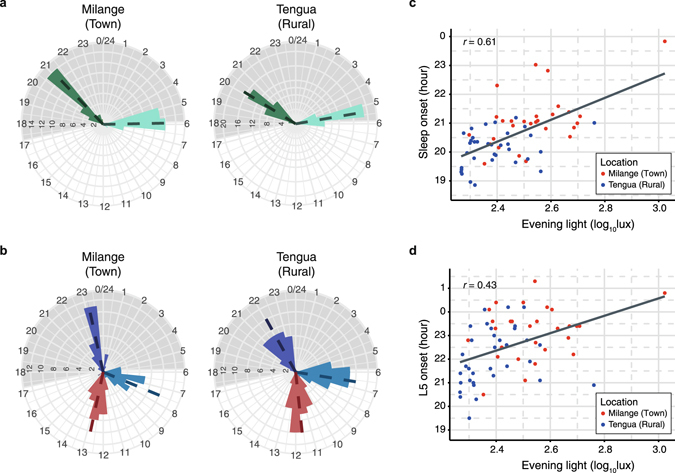
Phase of sleep and activity and amount of evening light exposure. (**a**,**b**) are circular histograms shown according to clock time where frequencies of temporal activity distributions are shown as distances from the centre. (**a**) Residents of Milange displayed a delayed onset of sleep (green) and a delayed wake up time (light green) compared to residents of Tengua. (**b**) Residents of Milange displayed a delayed M10 activity onset (light blue), a delayed L5 activity onset (purple) and a delayed centre of gravity for activity (red) relative to residents of Tengua. In (**a**,**b**) means (dotted lines) and histograms with 45 min bins (bars, counts on circumferences) are shown according to 24-h clock time (hours on radii). Grey shading represents average night duration. Sleep onset time (**c**) and L5 onset time (**d**) correlated with total exposure to light after sunset. Evening light exposure is distributed by location (Milange, red; Tengua, blue).

As expected for an electrified community, exposure to light after sunset was observed in Milange (Fig. 1b). Tengua, lacking electrification, showed significantly lower exposure to light in the three hours after sunset (Mann-Whitney-Wilcoxon, *W*(60) = 769, *P* < 0.001; Table 1). As this exposure period falls within the delay portion of the human phase response curve for light, we examined the phase of activity in Milange and Tengua. Tengua residents were significantly advanced relative to those of Milange, waking 32 ± 9 min earlier, beginning their most active 10-hour period (M10) 53 ± 20 min earlier, and with a centre of gravity of activity 73 ± 15 min earlier (*n* = 62; wake up time: Student’s *t*-test, *t*(60) = 3.78, *P* < 0.001; M10 onset: Welch’s *t*-test, *t*(60) = 2.58, *P* < 0.05; CG activity: *t*(60) = 4.78, df = 60, *P* < 0.001). In the evening, they had earlier onsets of sleep (59 ± 12 min) and of least active 5-hour period (L5) (67 ± 18 min) (*n* = 62; sleep onset: Mann-Whitney-Wilcoxon, *W*(60) = 789, *P* < 0.001; L5 onset: Student’s *t*-test, *t*(60) = 3.79, *P* < 0.001; Table 1, Fig. 2a,b). Sleep in both communities did not begin at the onset of darkness despite electric light being present only in Milange. Sleep onset was 3.8 h after sunset in Milange and 2.8 h after sunset in Tengua (Fig. 2b). The timing of the onset of sleep and of the least active 5 hours correlated with amount of evening light exposure (sleep onset: Pearson’s correlation, *r* = 0.61, *t*(60) = 5.93, *P* < 0.001, Fig. 2c; L5 onset: *r* = 0.43, *t*(60) = 3.65, *P* < 0.001, Fig. 2d) with more evening light resulting in later sleep onset time. Additionally, sleep onset and L5 onset correlated with amount of daytime light exposure M16, with more daytime light exposure correlating with an earlier sleep onset time (sleep onset: Pearson’s correlation, *r* = −0.41, *t*(60) = −3.49, *P* < 0.001; L5 onset: *r* = −0.23, *t*(60) = −2.58, *P* = 0.012, Supplementary Fig. S3). There was no difference between the communities in the regularity of the rhythm (IS), a measure of synchronisation with the zeitgeber (*n* = 62, Student’s *t*-test, *t*(60) = −1.10, *P* = 0.277; Table 1).

### Wake time and diurnal preference

Although both communities woke with or before sunrise, residents of Tengua rose 35 minutes before dawn and those of Milange just as the sun rose (Fig. 2a). The trend towards morningness in the Tengua community was reflected in the distribution of self-reported daily preference: 59% of the population prefer mornings over evenings or no preference compared to 38% of the Milange population (*n* = 74, Fisher’s exact test, *P* < 0.05; Supplementary Table S1). However, in Tengua, the most popular time indicated when they ‘felt at their best’ was the middle of the day (56%). This corresponded with the dip in daily activity (Fig. 1a), when most people rest after working in the fields. By contrast, most participants from Milange (51%) “felt at their best” in the mornings, despite 54% indicating no strong “preference” for mornings or evenings (*n* = 74, Fisher’s exact test, *P* < 0.05; Supplementary Table S1).

### Calculated measures of sleep

**Figure 3:**
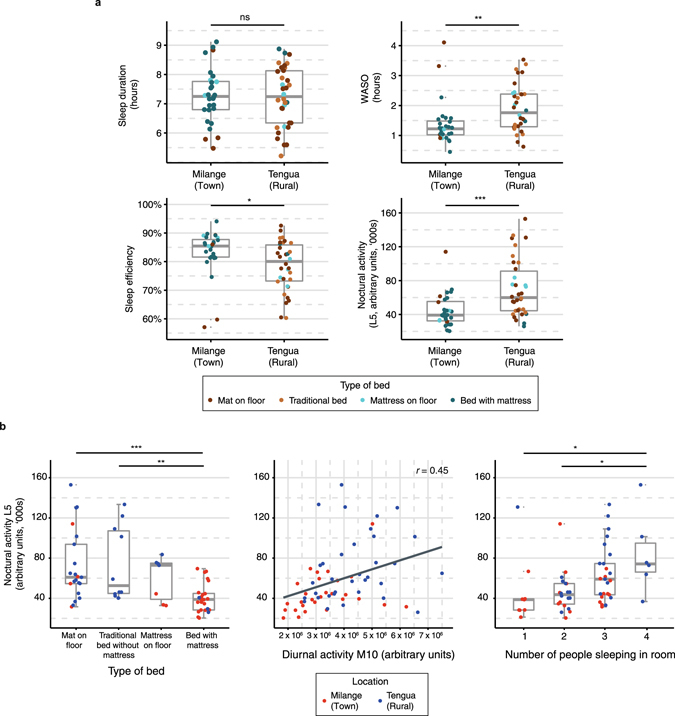
Sleep variables in town and rural settings. (**a**) Sleep variables by location. WASO, sleep efficiency and nocturnal activity L5 significantly differed between Milange and Tengua, though total sleep duration was not significantly different. Type of bed is represented by colour. ***P < 0.001; **P < 0.01; *P < 0.05, according to Table 1. (**b**) Environmental variables impact nocturnal activity L5. Type of bed (left), amount of diurnal activity M10 (centre) and number of people sleeping in the same room (right) associated with the amount of nocturnal activity across both locations (Milange, red; Tengua, blue). Significance between groups is denoted by Bonferroni corrected post-hoc tests, ***P < 0.001; **P < 0.01; *P < 0.05. Correlation (*r*) determined significant association between continuous variables.

Though the timing of activity and sleep differed between the town and rural zones, there was no difference in total sleep duration (*n* = 62, Student’s *t*-test, *t*(60) = 0.18, *P* = 0.860; Table 1, Fig. 3a). However, participants in Tengua had a significantly more fragmented rest-activity pattern, indicated by intradaily variability (IV) (*n* = 62, Student’s *t*-test, *t*(60) = 2.08, *P* = 0.041; Table 1). Furthermore, residents of rural Tengua had more disturbed sleep, with higher wake after sleep onset (WASO) (*n* = 62, Mann-Whitney-Wilcoxon, *W*(60) = 249, *P* = 0.001), a poorer sleep efficiency (*n* = 62, Mann-Whitney-Wilcoxon, *W*(60) = 652, *P* = 0.013), higher nocturnal activity L5 (*n* = 62, Student’s *t*-test, *t*(60) = −3.90, *P* < 0.001) and a greater number of nocturnal awakenings (*n* = 62, Student’s *t*-test, *t*(60) = −2.31, *P* < 0.05) than those of Milange (Table 1, Fig. 3a). The differences in sleep disturbance measures between locations remained after correction for age and sex in regression models, as higher sleep disturbance associated with the location of Tengua: (Linear regression, *n* = 62. WASO: adjusted *R* ^2^ = 0.25, *β*_location_ (standardised regression coefficient) = 0.22, *P* = 6.3 × 10^−5^; Sleep efficiency: adjusted *R* ^2^ = 0.21, *β*_location_ = −0.16, *P* = 0.0003; L5 nocturnal activity: adjusted *R* ^2^ = 0.29, *β*_location_ = 0.26, *P* = 6.5 × 10^−6^; N awakenings: adjusted *R* ^2^ = 0.25, *β*_location_ = 0.16, *P* = 0.0001).

### Determinants of qualitative measures of sleep

Potential reasons for poor sleep quality were compared between the two sites. There were significant differences in the distribution of maintenance of livestock, the type of bed on which people sleep, the number of people staying in the house and staying in the same room as the subject, and the amount of physical activity during the day (Table 1 and Supplementary Table S1). Across the whole population, the type of bed on which people sleep (see Supplementary Fig. S4 for more information) is the factor most strongly associated with calculated measures of sleep quality, as it significantly affects L5 nocturnal activity (*n* = 62, ANOVA, *F*(3, 58) = 7.74, *P* < 0.0002), WASO (ANOVA, *F*(3, 58) = 3.11, *P* = 0.033) and sleep efficiency (ANOVA, *F*(3, 58) = 3.33, *P* = 0.026; Fig. 3b). The amount of activity during the day also had a significant positive association with L5 nocturnal activity (Linear regression, *n* = 62, *R* ^2^ = 0.19, *β*_M10_ (standardised regression coefficient) = 0.67, *P* = 0.0002), WASO (*n* = 62, *R* ^2^ = 0.10, *β*_WASO_ = 0.48, *P* = 0.006) and sleep efficiency (*n* = 62, *R* ^2^ = 0.07, *β*_sleep efficiency_ = −0.080, *P* = 0.020). The number of people sleeping in the same room had a significant positive association with L5 nocturnal activity (*n* = 62, *R* ^2^ = 0.20, *β*_people in room_ = 0.086, *P* = 0.0002). Associations between L5 nocturnal activity and type of bed and the number of people sleeping in the same room remained significant when the regression models were adjusted for age, sex and location (*n* = 62. Type of bed: *R* ^2^ = 0.33, *β*_type of bed_ = −0.047, *P* = 0.042, *β*_location_ = 0.18, *P* = 0.016; People in room: *R* ^2^ = 0.39, *β*_people in room_ = 0.067, *P* = 0.002, *β*_location_ = 0.20, *P* = 0.0002). By contrast, L5 nocturnal activity was not affected by the amount of evening light in a regression model adjusted for age, sex and location (*n* = 62. M3 evening light: *R* ^2^ = 0.28, *β*_evening light_ = −5.6 × 10^−5^, *P* = 0.77, *β*_location_ = 0.25, *P* = 0.00005).

Despite significant differences in objective measures of sleep quality between each community, subjective measures did not differ. There were no significant differences in the distribution of responses to questions regarding sleep problems, i.e. difficulty initiating, maintaining and getting sufficient sleep, between the two groups (Fisher’s exact test, *n* = 74, initiation: *P* = 0.277; maintenance, *P* = 0.552; sufficient sleep, *P* = 0.115, Supplementary Table S1). Sleep maintenance and subjective quality problems were reported in over 30% of both populations, with Tengua displaying a higher proportion of sleep maintenance problems than Milange, but a higher proportion of the Milange population reporting tiredness once woken in the morning than Tengua (Supplementary Table S1). However, across the entire study population, there was no significant difference between the groups’ responses to questions regarding sleep problems, i.e. difficulty initiating, maintaining or getting sufficient sleep, and WASO, sleep efficiency or nocturnal activity (*n* = 62; WASO, initiation: *P* = 0.714, maintenance, *P* = 0.430, sufficient sleep, *P* = 0.156; sleep efficiency, initiation: *P* = 0.655, maintenance, *P* = 0.215, sufficient sleep, *P* = 0.150; L5, initiation: *P* = 0.752, maintenance, *P* = 0.240, sufficient sleep, *P* = 0.171).

### Summary

In summary, our results show that a town in transition to full urbanisation exhibited later, but not less, nor poorer quality, sleep than a rural community. In fact, sleep was more restless in the rural community, which was associated with a number of factors relating to a rural lifestyle. Rural residents typically had a larger proportion of participants sleeping on woven mats on the floor, a greater level of physical activity during the day and more people sleeping in the same room than urban residents. The pattern of exposure to, and synchronisation with, light differed between the two communities, with urban residents exposed to significantly more light at night than those in the rural community, and the rural community synchronising the majority of their activity with the hours of daylight.

## Discussion

Suggestions that we are experiencing a sleep deprivation epidemic have been associated with the change in balance of natural and artificial light exposure, made possible by the development and widespread use of electricity^3, 9^. One proposed impact of light at night in modern society is that it enables later bed times without a compensating extension of sleep in the morning, therefore reducing sleep duration^3^. Our analysis of two neighbouring communities, both of which share a cultural background, but only one of which is on the cusp of urbanisation, is consistent with the hypothesis that access to electricity delays the timing of sleep. However, here we find that the electrification of a town does not lead to a reduction in sleep duration, nor sleep quality, of its residents. Instead, our results suggest that some aspects of an urban environment and lifestyle may be beneficial to sleep.

Artificial light exposure at night, like that seen in Milange, is able to delay sleep through a combination of its direct, phase-delaying, effects on the circadian clock^14^ through its inhibitory effect on melatonin release^15, 16^, through its acute alerting action^17^, and through a behavioural effect by allowing people to perform activities that are not possible in darkness. Studies that have examined the effect of artificial light at night on human sleep timing and quality in “natural” environments confirm these laboratory observations. Access to electric light is associated with later sleep timing in hunter-gatherer Toba/Qom communities^8^, Amazonian rubber tappers^9^, adolescents in rural Brazil^18, 19^, and in intervention studies comparing the same participants with and without electric light^20^. Our results agree with these findings, with the delay in circadian and sleep timing in residents of Milange of up to one hour comparable to the delays recorded in previous actigraphy studies^8, 9, 20^. An interesting point to note is that the evening light in Milange is of relatively low brightness, often just a single, low power, tungsten filament bulb in a living room, quite unlike the high intensity illumination that is present in many industrialised households. Nevertheless, similar delays in sleep timing as that observed in Milange (around 1 h) have been measured in communities with comparably low levels of evening light exposure such as in the electrified Toba/Qom communities^8^. Interestingly, despite electricity only being present in Milange, both communities remained active after sunset and initiated sleep three to four hours after sunset. This is a similar pattern to that observed in hunter-gatherers by Yetish and colleagues^4^ and may reflect the use of candles and fires as light sources in Tengua, with the dim red wavelength-enhanced light having less of a biological effect than the bluer and brighter light emitted by electric bulbs^21, 22^.

In the same way, earlier sleep schedules are associated with greater exposure to bright light of more than 1000 lux^20^. Whilst both communities were exposed to substantially brighter light during the day than that recorded in industrialised societies^20^, residents of Tengua were exposed to significantly brighter light than those of Milange during daylight hours. As light in the late evening falls within the delaying portion of the human phase response curve to light, and light in the morning falls within the advancing portion^14^, it is likely that the differences in timing of activity and sleep in the two communities is a combination of advancing effects of the brighter light during the morning and the delaying effects of light at night. Nevertheless, despite differences in exposure to light, the regularity of the rest-activity rhythm with respect to the light dark cycle, IS, does not differ between the two communities. Bright light is a strong zeitgeber for the human circadian clock^14^, and here it appears that increased light at night in the context of very bright light exposure during the day is not sufficient to disrupt rhythm regularity, though it may have an effect on phase, as indicated by delayed activity in the town. For a conclusive investigation of the effect on the circadian clock of changing light exposure patterns during urbanisation, direct measurements of circadian phase such as dim-light melatonin onset would need to be conducted. In the developed world, changes in sleep timing and quality in urban environments associate with both a reduction in exposure to bright light during the day and an increase in light at night^23^. Our ability to control our lighting environments is therefore highly likely to have affected the timing, quality and duration of sleep in industrialised society.

Unlike in the Argentinian Chaco (the home of the Toba/Qom communities)^8^, the electrification of Milange was not associated with a shorter sleep duration, although it was associated with a shorter total sleep period. However, whilst an effect of different types of bedding cannot be excluded, these two community pairs may be difficult to compare, as the Chaco communities rely mostly on government subsidies whilst the people in the Milange district mostly sustain themselves by farming or small-scale commerce. Indeed, sleep duration in urban Milange was not significantly different to that of rural Tengua. In fact, both communities exhibiting durations slightly above the average of 7 h reported in industrialised societies such as the UK^24^ and similar to that seen in urbanising areas of Brazil^25, 26^. However, sleep was more disturbed and of lower calculated quality in rural Tengua, agreeing with the *developing economy sleep degradation hypothesis* put forward by Samson and colleagues^27^. Sleep patterns in societies are likely to be influenced by many additional factors associated with industrialisation and urbanisation, such as bed type and sleeping location, ambient temperature, number of individuals sharing a sleeping space, rearing of animals (including livestock), environmental noise, consumption of caffeine, the presence of television and the internet, and nature, hours, and location of occupation^28^. Ambient temperature presents particular considerations in a tropical climate, because of the need to consolidate both sleeping and working during the coolest hours of the 24-hour period. For a comprehensive understanding of sleep in humans, all these masking factors would need to be investigated. Our data revealed significant differences in the distribution of bed types, maintenance of livestock, number of people sleeping in the same room, and the intensity of physical activity as people move from rural to urban economies. Each of these factors is likely to have an impact on the quality of sleep. The actigraphy data indicated that the participants in Tengua (without electricity) move more during their sleep phase than those from Milange. This may be attributable to a larger proportion of the former (46%) sleeping on mats on the floor than those from Milange, where 76% reported sleeping on beds with mattresses. The greater movement during the rest period associates with sleeping on traditional beds (handwoven mats supported on a wooden or bamboo frame) and mats on the floor (Fig. 3b), and may be due to frequent changes in position because of discomfort. Reduced sleep quality is associated with lower back and shoulder pain^29, 30^ and, though muscle soreness was not directly measured in this study, it is possible that the greater level of physical activity measured in residents of Tengua, together with demanding nature of subsistence farming, may cause a substantial level of musculoskeletal pain that disrupts sleep quality, similar to that seen in Amazonian rubber tappers^30^. Furthermore, in rural parts of Mozambique, many people who raise livestock will keep them either in the house or in an enclosed backyard close to the house. In either case, nocturnal noises from the animals may affect the sleep of the inhabitants. Environmental noise has been shown to significantly disrupt sleep in an agricultural Malagasy community^27^, and is yet another factor associated with a rural environment that is likely to be detrimental to sleep. However, unlike that study, here we do not report a high degree of segmented sleep periods or napping in either community. It is possible that environmental noise is louder in the Malagasy community which may contribute to its shorter (6.5 h) and more disturbed (sleep efficiency 70.7%) sleep pattern^27^.

Even if we had the ability to directly observe “ancestral” human sleep habits and patterns, it is not likely that they would represent an optimum in terms of quality and quantity. Our species has, over a very long period, developed means of meeting other basic needs such as eating and transformed them from merely ensuring survival to sources of satisfaction and pleasure. Constructing buildings where we can sleep in safety and beds where we can sleep in comfort represents a similar development in meeting our homeostatic sleep drive, and in most societies, this development predates the availability of electricity by many generations. The results presented here complement the existing literature in important ways. The hunter-gatherer societies studied by Yetish and colleagues^4^ and Samson and colleagues^5^ exist near the edge of survival, and sleep in the open air. The rural community here, Tengua, is relatively more comfortable materially and lives in houses, which is apparent as the average sleep time in the Tengua community (7.23 h) was nearly one hour longer than the 6.4 h average in the populations studied by Yetish *et al*., who sleep on similarly basic objects such as mats. In the ethnically and culturally similar, neighbouring electrified community of Milange, average sleep duration was not significantly different (7.28 h). It appears that the only conclusion that can really be drawn about “ancestral sleep” from observations of communities at different stages between hunter-gatherers and post-industrial societies is that sleep duration is plastic in modern man and almost certainly was so in our ancestors as well. As shown both by the examples of hunter gatherers in the Southern hemisphere and large segments of the population in the Northern hemispheres, we can survive for long periods on less than optimal sleep, and end up doing so when activities of wakefulness are given higher priority. Our findings seem to suggest that the delay in sleep timing associated with electrification is quite a separate phenomenon from acquiring a daily habit of partial sleep deprivation.

Our observations from Milange confirmed the association between availability of artificial light after sunset and delayed sleep onset, though our findings do not support the hypothesis that artificial lighting in itself leads to a decrease in sleep time. In a majority of participants from Milange, the availability of electrical lighting was combined with more comfortable sleeping arrangements (above all, mattresses), and with less physically strenuous daytime activities. Thus, we certainly cannot conclude that electrification *increases* sleep time or quality. Rather, the data presented here illustrate the complexity of community-based sleep studies, involving light exposure, daytime activity, sleeping arrangements, and sources of nocturnal disturbances, and suggest that aspects of urban environments and lifestyles may be beneficial to sleep.

## Methods

### Study population

This work complied with the tenets of the Declaration of Helsinki and the study protocol was approved by the Faculty of Science Ethics Committee of the University of Cape Town (approval code FSREC 04–2016). A sample of adults was recruited from Milange district, Zambézia province, Mozambique. In the latest census data, the district has a population of 571,233, of which 93% lives in the rural zone and 7% in the principal town and trading centre, Milange (16.10°S, 35.76°E)^11^. 82.2% of the district’s population lives in mud brick or basic wooden houses, 86.9% of them with grass roofs and 94.5% with adobe or mud flooring^31^, 2% of the population has access to electricity, almost entirely in Milange which is the only settlement in the district with a connection to mains electricity^31^. From this district, 1 unelectrified, rural subsistence farming community (Localidade de Tengua: 16.23°S, 35.81°E) and 1 electrified, semi-rural market trading community approximately 30 km away (Milange: 16.10°S, 35.76°E) were selected with the agreement of local government officials. Maize growing is the dominant activity in Tengua, and domesticated animals include chickens, goats, pigs, pigeons and ducks. The two communities belong to the Marenje people group, a sub-population of the Makua language group of northern Mozambique^32^ and share a common culture with many family ties.

The participants comprised of 74 people of both sexes, 18 years and older, with an equal number from Milange (*n* = 37; aged 28.7 ± 6.6 years, mean ± SD) and Tengua (*n* = 37; aged approximately 39.1 ± 11.0 years) (See Supplementary Table S2 for further information). As many Tengua residents do not have official birth records, estimates of age were provided by the chief of the village. Temporary migrants and any participant taking chronic medication, including antidepressants and beta-blockers, were not included. Recruitment took place between April and June 2016. All participants were informed as to the purpose of the study and the testing to be undertaken. On agreeing to participate, all participants were asked to sign a consent form, or provided recorded verbal consent. Participants were not rewarded financially for taking part; participation was completely voluntary.

Precise latitude and longitude coordinates of the locations of sample groups were determined with MyTracks (Google, Mountain View, CA), and Google Earth. The National Oceanic & Atmospheric Administration Solar Calculator (http://www.esrl.noaa.gov/gmd/grad/solcalc/) was used to determine the times of sunrise, sunset and solar noon at each site on the 7th day of the collection window at each of the sites. Average sunrise was at 05:55, average sunset was at 17:18 and average solar noon was at 11:36 local time (See Supplementary Table S2 for further information).

### Sleeping environment and preference questionnaire

To investigate preference for morning or evening activity, prevalence of insomnia-like symptoms, and sleep environments, interviews were conducted with each participant with the assistance of a Milange-based community development field worker. A questionnaire was derived from a similar study conducted in Haiti^1^ which took into account the low levels of literacy and low usage of watches or clocks, and asked each participant about their household environment (including the total number of people living in the dwelling and the total number of people sleeping in the same room as the participant), what the participant slept on, whether the participant kept animals, and questions about daily preference, sleep initiation and sleep maintenance problems (See Supplementary Information). The questionnaire was translated into the local languages (Portuguese and Chichewa), validated by Portuguese- and Chichewa-speaking colleagues of ADB and LCR, and administered by ADB and the field worker.

Morning was defined as the period between rising and 10:00 (approximately 4 hours after dawn), evening was defined as the period from 16:00 until bed. Answers of “yes” to questions with “Do you have any difficulty/Do you have any problems/Do you sometimes” signified it was usual each week, while answers of “sometimes” signified it occurred with a monthly frequency, answers of “no” signified a less than monthly frequency.

### Actigraphy

To investigate rest-activity rhythms, sleep quantity and quality, and light exposure, continuous actigraphy was carried out with each participant for a two-week period using a wrist actigraphy device which contained accelerometer, light and temperature sensors (ActTrust AT0503, Condor Instruments, São Paulo, SP, Brazil). The devices recorded activity, environmental light (infrared, red, blue and green light), environmental temperature and skin temperature using 60-s epochs. Compliance among the study population was reasonable, with 11 of the 74 participants (9 from Milange and 2 from Tengua) excluded from the analysis failing to wear the watch for a sufficient continuous recording period and 1 participant excluded for instrument failure. Representative actograms are shown in Supplementary Figure S5.

Data were subject to non-parametric analysis^33^ to find the following diurnal characteristics: total activity in the most active ten-hour period (M10) which reflects diurnal activity; total activity in the least active five-hour period (L5) which reflects nocturnal activity; total activity in the 3 hours after sunset (M3 evening activity) which reflects post-sunset activity; the onset of the 10 hours of most activity (M10 onset) which reflects wake up time; the onset of the 5 hours of least activity (L5 onset) which reflects the inactive period; the centres of gravity of activity (CG activity) and light (CG light) which reflect the acrophases of activity and light respectively; Intradaily variability (IV) which provides information on rest-activity rhythm fragmentation; interdaily stability (IS) which yields information about the rest-activity rhythm synchronisation with the light-dark cycle; total light exposure in the 16 hours with most illumination (M16 light) which reflects the quantity of light exposure during the day; total light exposure in the 3 hours after sunset (M3f) which reflects light at night. Phase angle is presented as the difference (in min) between CG activity and CG light.

Sleep characteristics were calculated using the Cole-Kripke algorithm^34, 35^ in ActStudio (version 1.0.5.0, Condor Instruments) to calculate sleep onset time; wake up time; total sleep period, which reflects the total period of inactivity scored by ActStudio and is equivalent to the interval between sleep onset and offset; total sleep duration, which is the total duration of ActStudio-scored sleep within the total sleep period; wake after sleep onset (WASO) which reflects the amount of time spent awake between the onset of sleep and wake up; and sleep efficiency, which reflects the percentage of the period of inactivity that a participant spent asleep. Each record was visually inspected to identify and exclude instances of artefact. Since both Milange and Tengua have high rates of illiteracy, sleep diaries were unable to be used in the analysis of sleep.

### Statistical analysis

Data are presented throughout as mean ± SEM. Data for M10, L5, wake up time, total sleep period and number of awakenings variables were normalised by log transformation before statistical analysis. Student’s *t*-tests, Welch’s *t*-tests and Mann-Whitney-Wilcoxon tests were used to test differences in rest-activity and sleep variables between Tengua and Milange, with Student’s *t*-tests and Fisher’s exact tests used to test differences in responses to the questionnaire. One-way ANOVA or Kruskal-Wallis tests were used to test differences in sleep quality variables according to covariate data. Univariate and multiple regression analysis were used for predicting the associations between rest-activity/sleep measures and covariate data. The level of significance was set at *P* = 0.05. The statistical analysis was carried out using the R project for statistical computing (www.r-project.org).

## Electronic supplementary material

Supplementary information
