# Supplementary material for: Comparison between an African town and a neighbouring village shows delayed, but not decreased, sleep during the early stages of urbanisation
Source: Sci Rep. 2017 Jul 18;7:5697. doi: 10.1038/s41598-017-05712-3 (PMC5515911; doi:10.1038/s41598-017-05712-3)
Supplement: Supplementary file 1 — Supplementary information [file 41598_2017_5712_MOESM1_ESM.pdf]

Supplementary Information

Comparison between an African town and a neighbouring village shows delayed, but not decreased, sleep during the early stages of urbanisation

Andrew D. Beale, Mario Pedrazzoli, Bruno da Silva B. Gonçalves, Felipe Beijamini, Núbia E. Duarte, Kieren J. Egan, Kristen L. Knutson, Malcolm von Schantz, Laura C. Roden

Supplementary Methods

The questionnaire was designed to ask participants about their sleep environment, their preference for morning or evening, their subjective view on their sleep quality, and considered the low levels of literacy and low usage of watches or clocks. The questionnaire was conceived in English and translated and validated in both Portuguese and Chichewa, using appropriate vocabulary for the region. It was administered in Portuguese or Chichewa according to the participant's preference, which tended to be more Portuguese in Milange and more Chichewa in Tengua. Below is the text used, with the English question in bold type, Chichewa in standard type and Portuguese in italics.

Questionnaire/Mafunso/Questionário

1. How many people are staying/living in your house including you?
Chiwerengero cha anthu amene amakhala m'nyumba mwanu (kuphatikizapo inu) ndi angati?
Quantos pessoas estão a morar na sua casa incluindo você?

2. How many rooms does your house have?
Nyumba yanu ili ndi zipinda zingati?
A sua casa tem quantos divisões?

3. How many people sleep in the same room as you, including you?
Ndi anthu angati amene amagona mu chipinda chimodzi ndi inu (kuphatikizapo inu)?
Quantos pessoas estão a dormir no mesmo quarto do que você incluindo você?

4.

a) **Do you keep livestock?**
Mumasunga ziweto?
Você mantém animais da fazenda?

b) **Mention the type of livestock you keep**
Tchulani mtundu wa ziweto zimene mumasunga
Mencione que tipos dos animais da fazenda que você mantém

c) **Do the livestock sleep in your house?**
Kodi ziweto zimenezi zimagona mnyumba mwanu?
Os animais da fazenda dormem na sua casa?

d) **How many livestock sleep in your house?**
Mumagona ndi ziweto zingati mnyumba mwanu?
Quantos animais da fazenda dormen na sua casa?

e) **Do the livestock sleep in the same room as you?**
Kodi ziweto zimagona mu chipinda chimodzi ndi inu?
Os animais da fazenda dormen no mesmo quarto do que você?

f) **How many livestock sleep in the same room as you?**
Ndi ziweto zingati zimene zimagona mu chipinda chimodzi ndi inu?
Quantos animais da fazenda dormen no mesmo quarto do que você?

5. What do you sleep on?
Kodi mumagona pa chani kunyumba kwanu?
Em que é que você dorme?

- **On a mat, on a floor**
  pa mphasa pansi
  na esteira, no chão
- **On a matress only**
  pa matress okha
  somente no colchão
- **On a bed**
  pa kama
  na cama

6. Do you sleep in a mosquito net?
Kodi mumagona mu ukonde pozitetedza ku udzudzu?
Você dorme dentro duma rede mosquiteira?

7. Do you prefer mornings or evenings or anytime?
Você prefere as manhãs ou as tardinhas ou qualquer hora?
Kodi mumakonda m'mawa, kapena madzulo, kapenanso nthawi iliyonse?

8. The time you just gave, is it because it is the time when you feel your best, or it is when you are most productive at at work?
Yankho lomwe mwasankhalo, ndichifukwa choti ndi nthawi imene mumamva bwino, kapena ndi nthawi imene mumatanganidwa ku nchito
Essa resposta que você escolheu, é porque é o horário quando se sente melhor, ou porque é um horário quando está mais produtivo no trabalho?

9. At what time of day do you feel your best?
Ndinthawi iti ya patsiku mumamva bwino?
Em qual período do dia você se sente o seu melhor?

- **morning**
  mmawa
  de manhã
- **midday**
  masana
  Ao meio-dia
- **evening**
  madzulo
  À tardinha ou no final da tarde
- **any time**
  nthawi iliyonse
  qualquer hora

10. Do you have any difficulty falling asleep normally?
Kodi inu mumavutika kuti tulo tibwere?
Normalemente, você tem qualquer dificuldade para adormecer?

11. Do you have any difficulty staying asleep for a long time normally?
Kodi inu mumavutika kukhala mtulo nthawi yayitali?

Normalemente, você tem qualquer dificuldade de permanecer adormecido por um longo tempo?

12. Do you have any problems waking up too early normally?
Kodi muli ndi vuto lakudzuka mmawa kwambiri?
Normalemente, você tem quaisquer problemas para acordar muito cedo?

13. Do you sometimes wake up still feeling tired?
Nthawi zina mumadzuka mukadali otopa?
Às vezes você acorda ainda sentindo cansado/cansada?

Supplementary Data

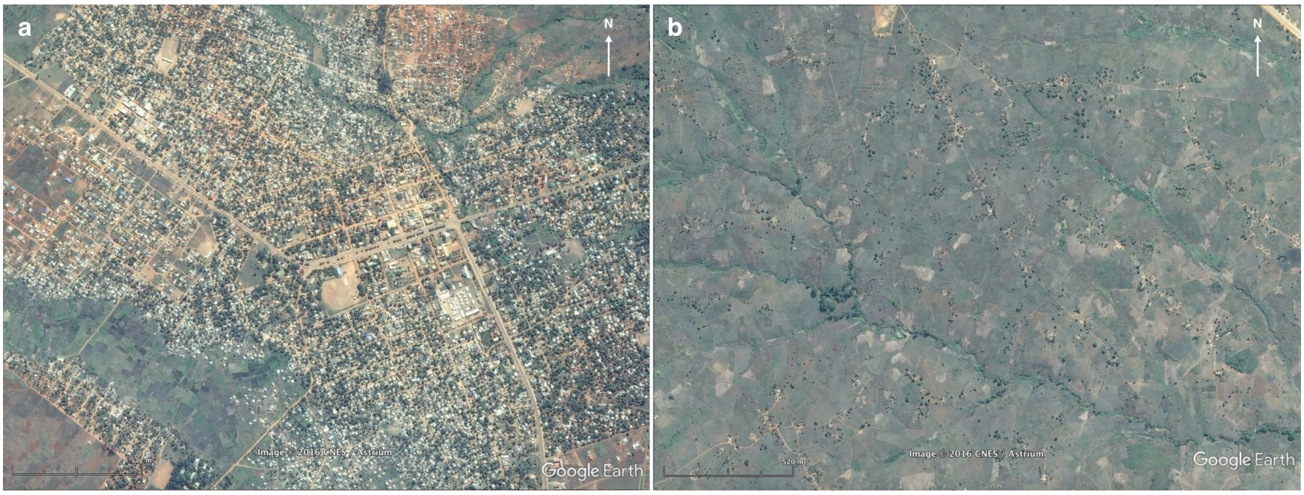

Supplementary Figure S1. Satellite imagery comparing **a** Milange and **b** Tengua at 3km eye altitude. **a,** The principal avenue of the town can be seen running in a v shape from the top left south-east to the centre of the image before turning west towards the right-hand side of the image. **b,** Viewed from the same altitude, the rural community of Tengua is sparsely populated. Formal roads are absent. Instead, the community is crossed with numerous foot paths linking dwelling to dwelling and to farmland. Scale bars in **a** and **b** represent 512 m and 520 m respectively. Imagery date 19/08/2016, data © 2016 Google, CNES/Astrium.

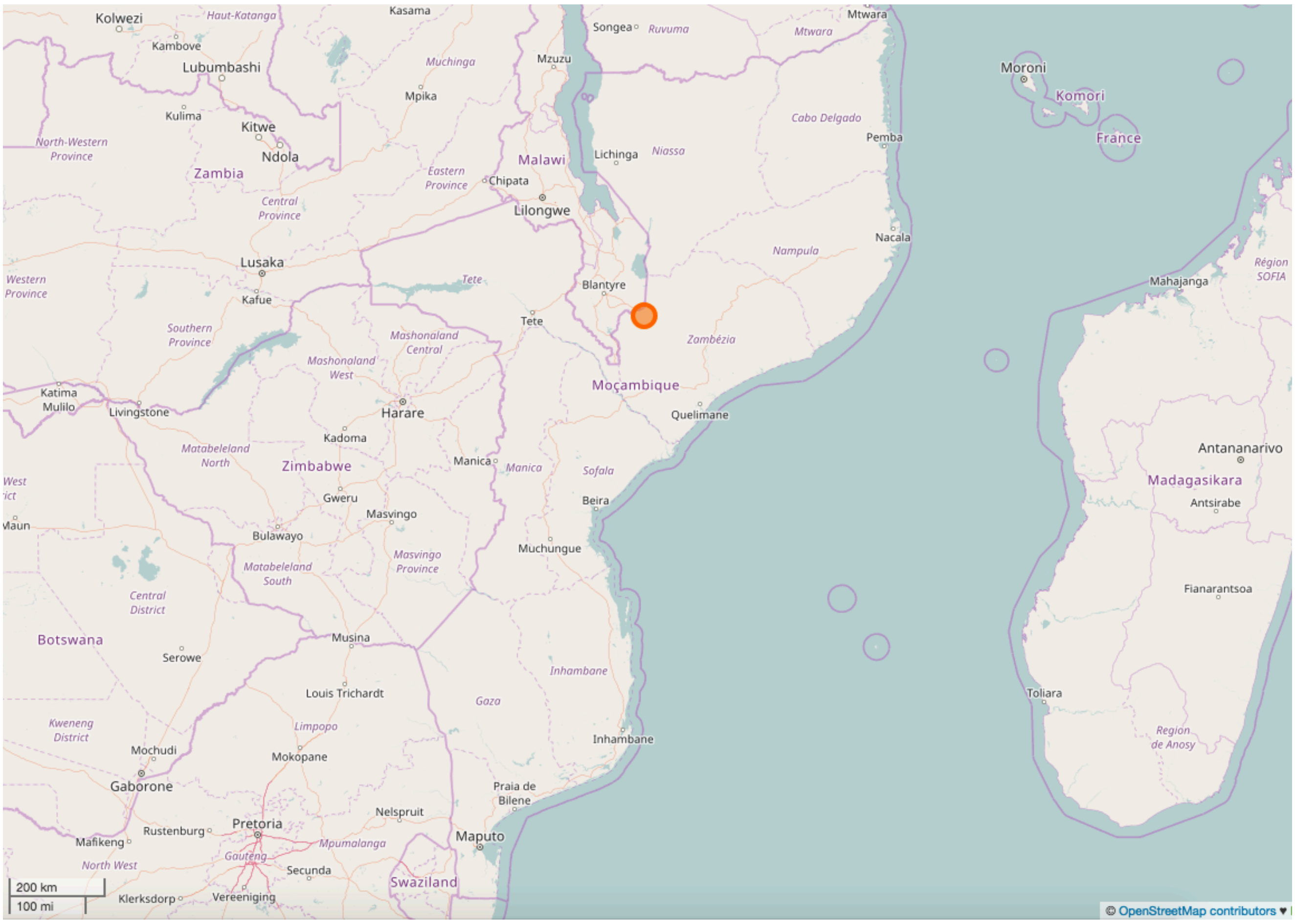

Supplementary Figure S2. Map of Mozambique with the study area of highlighted (orange). Map data © OpenStreetMap contributors, accessed 12th February 2017. Map data is available under the Open Database Licence.

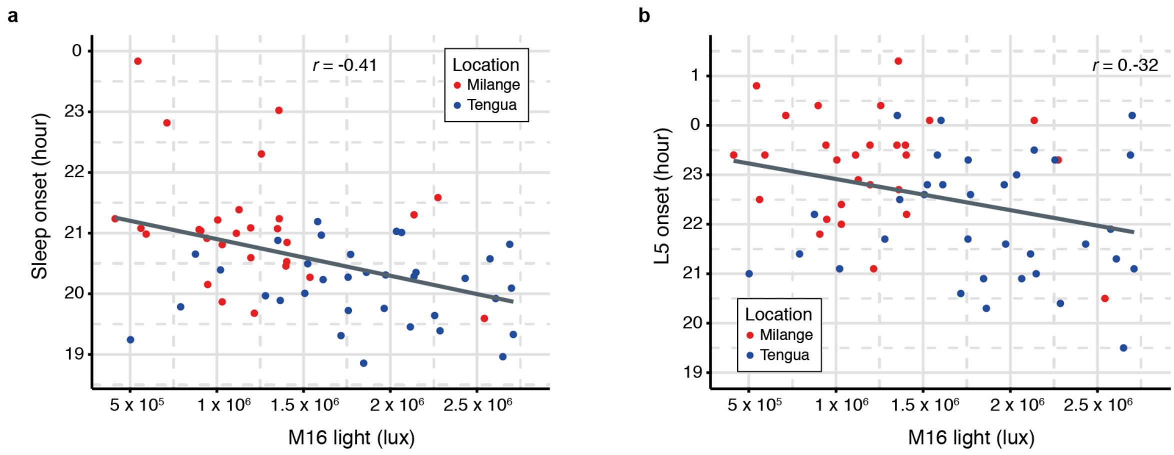

Supplementary Figure S3. Sleep onset time **a** and L5 onset time **b** are correlated to total exposure to light in the brightest 16 hours of the day (M16). M16 is distributed by location (Milange town, red; Tengua rural, blue).

a

c

b

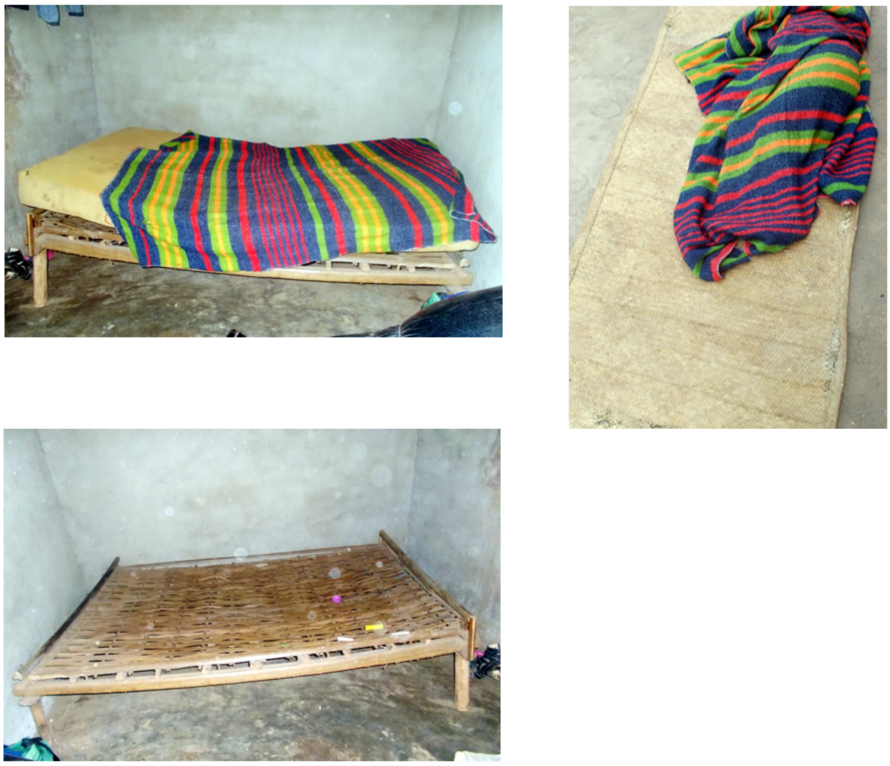

Supplementary Figure S4. Examples of the types of bed recorded in the study. **a,** Wooden or bamboo bed frame with a simple foam mattress for sleeping on. **b,** Traditional bed, which consists of a handwoven mat supported on a wooden or bamboo frame. **c,** Handwoven grass mat, placed directly on the ground as shown in the photograph. Not shown is the fourth category of bed type recorded in the study, mattress on the floor, which is a simple foam mattress as seen in **a**, but placed directly on the floor with no bed frame. Images courtesy of Micael Jose Calimba under an Open Access Licence.

Milange 22 M

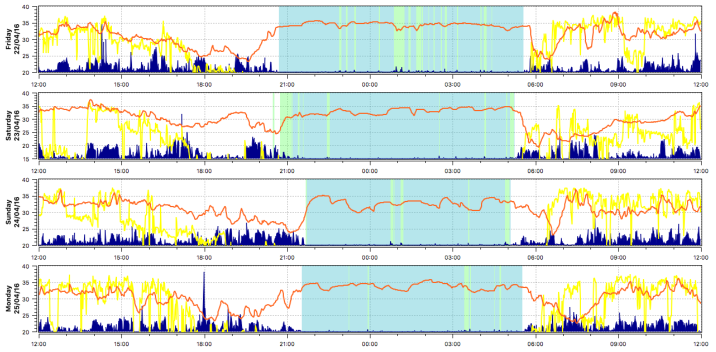

Milange 38 M

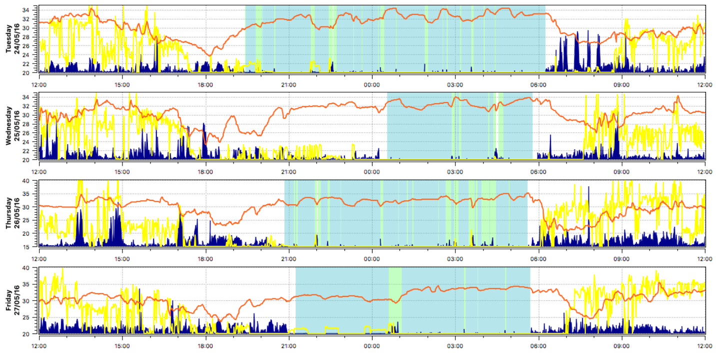

Tengua, 32 M

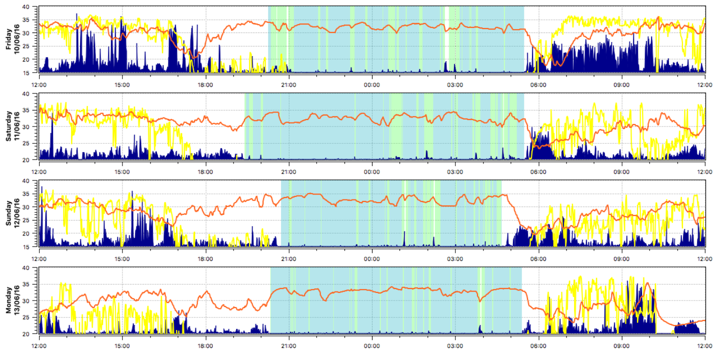

Tengua, 34 F

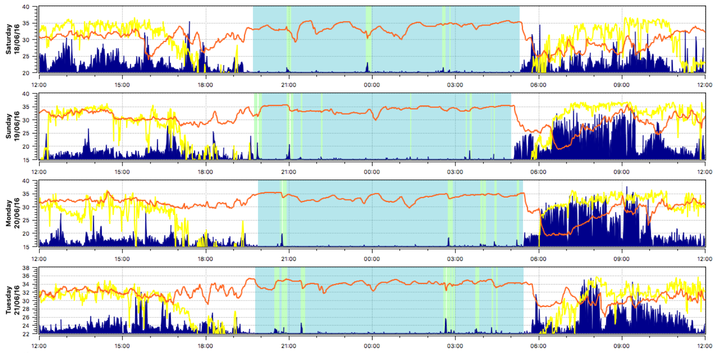

Supplementary Figure S5. Representative actograms from both communities. Yellow, light level; blue, number of movements in each 60-s interval; orange, temperature at wrist; light blue, ActStudio-scored sleep; light green, ActStudio-scored rest. Sleep duration is defined as ActStudio-scored sleep time (light blue). Total sleep period is defined as the interval between sleep onset and sleep offset, and is equal to sleep duration plus wake after sleep onset (WASO).

		Milange		Tengua		
		mean n = 37	SEM	mean n = 37	SEM	p
How many people are staying in your house including you?		5.78	0.47	6.00	0.36	0.716[a]
How many rooms do your house have?		4.16	0.28	3.14	0.20	0.004[a]
How many people sleep in the same room as you including you?		2.19	0.14	2.95	0.19	0.002[a]
	**Categories**	**n**	**%**	**n**	**%**	
Do you keep livestock?	Yes	8	22%	28	76%	
	No	29	78%	9	24%	6.15E-06[b]
Do the livestock sleep in your house?	Yes	4	50%	2	7%	
	No	4	50%	26	93%	0.014[b]
Do the livestock sleep in the same room as you?	Yes	0	0%	1	50%	
	No	4	100%	1	50%	0.286[b]
What do you sleep on?	Mat on floor	5	14%	17	46%	
	Mattress on floor	4	11%	7	19%	
	Traditional bed without mattress	0	0%	10	27%	
	Bed with mattress	28	76%	3	8%	1.33E-09[b]
Do you sleep in a mosquito net?	Yes	31	84%	23	62%	
	No	6	16%	14	38%	0.065[b]
Do you prefer mornings or evenings or neither?	Morning	14	38%	22	59%	
	Neither	20	54%	8	22%	
	Evening	3	8%	7	19%	0.013[b]
At what time of day do you feel your best?	Morning	19	51%	9	33%	
	Middle of the day	5	14%	15	56%	
	Evening	9	24%	0	0%	
	No preference	4	11%	3	11%	0.032[b]
Do you have any difficulty falling asleep normally?	Yes	7	19%	12	32%	
	Sometimes	5	14%	2	5%	
	No	25	68%	23	62%	0.277[b]
Do you have any difficulty staying asleep for a long time normally?	Yes	11	30%	14	38%	
	Sometimes	3	8%	5	14%	
	No	23	62%	18	49%	0.552[b]
Do you have any problems waking up too early normally?	Yes	7	19%	6	16%	
	Sometimes	3	8%	0	0%	
	No	27	73%	31	84%	0.231[b]
Do you sometimes wake up still feeling tired?	Yes	17	46%	11	30%	
	Sometimes	9	24%	6	16%	
	No	11	30%	20	54%	0.115[b]

Supplementary Table S1. (Previous page). Summary of responses to the questionnaire. [a] = Student's t-test, [b] = Fisher's exact test.

Group	Start	Finish	Latitude	Longitude	Sunrise	Sunset	Solar noon	Night duration (h)	Total participants	Number of women	Age ± s.d. (years)	Total days
M1	12/04/16	26/04/16	-16.1	35.8	05:46	17:26	11:36	12:20	15	8	27.4±5.7	203
M2	28/04/16	12/05/16	-16.1	35.8	05:49	17:18	11:33	12:31	15	7	27.2±7	203
M3	14/05/16	28/05/16	-16.1	35.8	05:54	17:13	11:33	12:41	7	3	34.7±4.7	97
T1	17/05/16	30/05/16	-16.2	35.8	05:55	17:12	11:33	12:43	8	1	36.0±16.4	104
T2	31/05/16	14/06/16	-16.3	35.9	06:00	17:11	11:35	12:49	14	3	39.9±8.1	196
T3	17/06/16	30/06/16	-16.4	36.0	06:04	17:13	11:38	12:51	15	9	39.9±10.5	195
								Total	74	31		998

Supplementary Table S2. Information about participant groups. Due to the absence of official birth records, ages in Tengua are estimates provided by the chief of the village.
